# Immunomodulator comedication promotes the reversal of anti-drug antibody-mediated loss of response to anti-TNF therapy in inflammatory bowel disease

**DOI:** 10.1007/s00384-023-04349-1

**Published:** 2023-02-25

**Authors:** Johannes Stallhofer, Jan Guse, Miriam Kesselmeier, Philip Christian Grunert, Kathleen Lange, Robert Stalmann, Verena Eckardt, Andreas Stallmach

**Affiliations:** 1 Department of Internal Medicine IV (Gastroenterology, Hepatology and Infectious Diseases), Jena University Hospital, Am Klinikum 1, Jena, 07747 Germany; 2 Institute of Medical Statistics, Computer and Data Sciences, Jena University Hospital, Jena, Germany; 3 Institute of Clinical Chemistry and Laboratory Diagnostics, Centralized Diagnostic Laboratory Services, Jena University Hospital, Jena, Germany

**Keywords:** Inflammatory bowel disease, Anti-TNF therapy, Loss of response, Anti-drug antibodies, Immunomodulator combination therapy

## Abstract

**Purpose:**

Loss of therapeutic response (LOR) due to anti-drug antibodies (ADA) against tumor necrosis factor (TNF) inhibitors is common in patients with inflammatory bowel disease (IBD). We aimed to investigate whether immunomodulator comedication can reverse the immunogenic LOR to TNF inhibitors in IBD.

**Methods:**

In this real-world retrospective cohort study, 123 IBD patients with neutralizing ADA to infliximab or adalimumab and concomitant subtherapeutic trough levels were screened for clinical LOR. Subsequent ADA and trough level measurements and clinical outcomes were analyzed for patients who received either immunomodulator comedication or dose intensification of infliximab or adalimumab to overcome LOR.

**Results:**

Following immunogenic LOR, the initial anti-TNF regimen was optimized in 33 patients. In univariable and multivariable logistic regression analyses, immunomodulator comedication was identified as the crucial factor for regaining clinical remission and ADA clearance. Detectable trough levels (≥ 0.98 or ≥ 1.00 mg/L, respectively) had optimal predictive performance for both endpoints in receiver operating characteristics curves [area under the curve 0.86 (95% confidence interval 0.68–1.00) for regaining clinical remission, 0.87 (0.71–1.00) for ADA clearance]. Furthermore, 11/20 patients (55%) on a comedication with azathioprine or methotrexate and 2/13 patients (15%) receiving anti-TNF dose intensification exclusively (*P* = 0.032) exhibited ADA elimination, regain of therapeutic trough levels, and clinical remission. Regain of clinical remission alone was achieved in 17/20 (85%) patients receiving comedication and 2/13 (15%) patients receiving anti-TNF dose intensification (*P* = 1.6 × 10^−4^).

**Conclusion:**

Immunogenic LOR to infliximab or adalimumab in IBD can be successfully reversed using immunomodulator comedication.

**Supplementary Information:**

The online version contains supplementary material available at 10.1007/s00384-023-04349-1.

## Introduction

The two major types of inflammatory bowel disease (IBD), namely Crohn’s disease (CD) and ulcerative colitis (UC) affect millions of people worldwide [1]. The incidence and prevalence of these diseases are increasing globally. It is expected that by 2030, approximately 1% of the population in industrialized countries will be affected by IBD, which will significantly affect the patient’s quality of life and increase the burden on healthcare systems [1]. With the development of monoclonal antibodies against tumor necrosis factor (TNF)-α, the management of moderate-to-severe IBD was revolutionized about two decades ago [2]. Anti-TNF therapy prolongs steroid-free clinical remission, promotes mucosal healing, facilitates closure of fistulas in CD [3], prevents complications, and improves the patient’s quality of life [2]. Despite the development of newer biologics with different modes of action, the two anti-TNF agents infliximab (IFX) and adalimumab (ADL) remain first-line biological therapeutics for CD [4] and UC [5].

Approximately two-thirds of patients initially respond to anti-TNF treatment; however, a large proportion of responders experience a secondary loss of response (LOR) over time [6, 7]. Systematic reviews of 16 IFX [8] and 39 ADL studies [9] revealed that the annual risk for secondary LOR is 13% and 20% per patient-year, respectively. Within a year of anti-TNF treatment initiation, up to 50% of primary responders lose their ability to respond [6, 10].

Besides mechanistic failure, both primary nonresponse and secondary LOR can be due to undetectable or subtherapeutic drug concentrations with or without the development of anti-drug antibodies (ADA) [6]. ADA can bind to and form complexes with the circulating biologic, thereby, preventing its therapeutic effect [11]. Up to 63% of IFX-treated patients and 29% of ADL-treated patients develop ADA within the first 54 weeks of therapy [7]. A previously reported large prospective personalized anti-TNF therapy in Crohn’s disease study (PANTS) identified smoking, obesity, suboptimal week 14 drug concentrations, and the absence of a co-immunomodulator as additional risk factors for ADA production [7].

Accumulating evidence suggests that the presence of an immunomodulator can prevent the formation of ADA [12]. However, it remains unknown whether immunomodulator co-administration suppresses ADA production and increases the therapeutic drug levels to promote clinical remission in patients with ADA-associated LOR and subtherapeutic drug levels. So far, this issue has been investigated only in a few case series, small studies involving 5–23 adults with IBD [13–17], and one study in pediatric IBD [18]. Results of these studies suggest that combination therapy with azathioprine or methotrexate can reverse ADA-mediated LOR. However, in these studies, the included adult patients almost exclusively suffered from CD. Ben-Horin et al. [13] and Strik et al. [16] included only one patient with UC while Ungar et al. [15] had two UC patients.

Generally, in ADA-mediated immunogenic LOR with subtherapeutic drug levels, a switch from IFX to ADL or vice versa, or a switch to another drug is standard clinical practice [19]. However, both primary and secondary nonresponse to the first anti-TNF agent is associated with a lower likelihood of clinical remission with the subsequent anti-TNF antibody [20]. Previous studies showed that prior immunogenicity could predict the development of ADA against a second anti-TNF agent [12, 21, 22]. Furthermore, out-of-class therapeutic options for anti-TNF-experienced patients are not promising, and as compared to anti-TNF naïve patients, they usually exhibit lower clinical remission rates with second-line therapies [23].

Hence, optimization of the existing anti-TNF therapy is crucial. Therefore, we aimed to investigate whether combination therapy with azathioprine or methotrexate can be used to overcome the immunogenic LOR to infliximab or adalimumab. We retrospectively analyzed the real-world data from our large university IBD database over a period of 8 years. IBD patients who received dose intensification for the anti-TNF agent alone served as the comparison group.

## Materials and methods

### Patient screening, inclusion criteria, and endpoints

In this real-world retrospective cohort study, we performed a database search for all IFX and ADL trough levels and all ADA against IFX and ADL that were measured in adult IBD inpatients and outpatients between January 1, 2013 and December 31, 2020 at Jena University Hospital (Jena, Germany). Patients with subtherapeutic trough levels (IFX < 3 mg/L, ADL < 5 mg/L) and concomitantly elevated ADA (IFX-ADA ≥ 10 U/mL, ADL-ADA ≥ 10 U/mL) in the same blood sample at a variable time point T1 were further screened for the presence of clinical LOR (see below for definition) by a thorough review of patient charts. The coincidence of a pharmacokinetic LOR (subtherapeutic trough levels along with elevated ADA) and a clinical LOR was termed immunogenic LOR. For patients with an immunogenic LOR, the details of therapeutic interventions were retrieved from the medical records. Patients who switched to another therapy or underwent surgery did not qualify for this analysis and were excluded. Patients whose initial IFX or ADL therapy was optimized after immunogenic LOR were included in the analysis. All subsequent IFX or ADL trough level and ADA level measurements were examined along with clinical outcomes. The timing of serological evaluation was up to the treating physician and therefore variable. The endpoints were (i) regain of clinical remission, (ii) ADA clearance, and (iii) regain of clinical remission along with the therapeutic response (see below for definitions) at a variable time point T2. In cases with multiple follow-up measurements, the time of the last concomitant ADA and trough levels measurement for an individual patient was considered T2. Data were collected until September 30, 2021.

Clinical LOR was defined by a Harvey-Bradshaw Index (HBI) ≥ 5 for CD [24] and a partial Mayo score (PMS) ≥ 2 for UC [25] in conjunction with the decision of the treating physician to change the therapy. In the absence of symptomatic LOR, if the treating physician decided to change the therapy because of a fecal calprotectin level > 250 µg [26] (surrogate parameter of active intestinal inflammation), or a simple endoscopic score for CD (SES-CD) > 2 [27], or an endoscopic Mayo subscore (EMSS) > 1 for UC [28], this was also considered as a clinical LOR.

The initial IFX or ADL therapy was deemed optimized if the dose remained unchanged/increased in combination with azathioprine or methotrexate (immunomodulator combination therapy group) or intensified with no immunomodulator comedication (anti-TNF dose intensification group). The immunomodulator comedication was either newly initiated, dose escalated, or continued.

Regain of clinical remission was defined as HBI < 5 for CD [24] or PMS < 2 for UC [25] for patients with symptomatic LOR, or a fecal calprotectin level ≤ 250 µg/g feces or documented endoscopic remission (SES-CD ≤ 2, EMSS ≤ 1) for patients with an isolated biochemical or endoscopic LOR. A pharmacokinetic response was defined as the complete clearance of ADA in combination with an increase of trough levels to therapeutic levels (IFX ≥ 3 mg/L, ADL ≥ 5 mg/L).

### Laboratory measurements

IFX and ADL trough levels were quantified using the enzyme-linked immunosorbent assay (IDKmonitor^®^ Infliximab Drug Level ELISA and IDKmonitor^®^ Adalimumab Drug Level ELISA kits; Immundiagnostik AG, Bensheim, Germany) during routine diagnostics. The timing of the evaluation was clinically decided. In most of the samples, IFX-ADA and ADL-ADA levels (554 of 730, 76%) were determined using the drug-tolerant enzyme-linked immunosorbent assay (IDKmonitor^®^ Infliximab total ADA ELISA and IDKmonitor^®^ Adalimumab total ADA ELISA kits; Immundiagnostik AG, Bensheim, Germany), which can detect free as well as drug-bound antibodies. In remaining samples (176 of 730, 24%), ADA levels were measured using the drug-sensitive enzyme-linked immunosorbent assay (IDKmonitor^®^ Infliximab free ADA ELISA and IDKmonitor^®^ Adalimumab free ADA ELISA kits; Immundiagnostik AG, Bensheim, Germany), which can detect free ADA.

Fecal calprotectin level was measured using the enzyme-linked immunoassay (IDK^®^ Calprotectin ELISA kit; Immundiagnostik AG, Bensheim, Germany). The routine measurement of C-reactive protein (CRP) levels was performed using the turbidimetric latex immunoassay on the ARCHITECT system (MULTIGENT CRP Vario, Abbott GmbH, Wiesbaden, Germany) from January 2013 until May 2020 and on the Cobas^®^ system (Tina-quant C-Reactive Protein IV, Roche Diagnostics GmbH, Mannheim, Germany) from June 2020 onwards.

### Statistical analyses

Continuous variables are presented as median with first and third quartiles (Q1, Q3) and categorical variables as absolute numbers and relative frequencies (*n*, %). Groups were compared using the Mann–Whitney *U* test (unpaired test) or Wilcoxon signed rank test (paired test) for continuous variables and Fisher’s exact test for categorical variables. To investigate the association of the endpoints (i) regain of clinical remission, (ii) ADA clearance, and (iii) regain of clinical remission along with a pharmacokinetic response with the immunomodulator combination therapy, univariable and multivariable Firth’s logistic regression modeling was performed. All multivariable models were adjusted for age and other possible confounders. The investigated independent variables were selected based on clinical judgment. Regression analysis results are reported as adjusted odds ratio (OR) and the corresponding 95% profile-likelihood confidence interval (CI). To assess the diagnostic abilities of trough levels, ADA levels, and CRP levels at the time point T1 for the outcomes (i)–(iii), empirical receiver operating characteristics (ROC) curve analyses were performed and the area under the curve (AUC) was determined for both treatment groups separately. The Youden index was used to identify an optimal cut-off value for trough levels, ADA levels, and CRP levels, to predict the likeliness of treatment success in patients who received immunomodulator combination therapy and of treatment failure in patients who received anti-TNF dose intensification exclusively. All reported *P*-values are two-sided and a significance level of 0.05 was considered. Logistic regression and ROC analyses were performed using R (version 4.0.3) including the R packages logistf (version 1.24.1) and ROCit (version 2.1.1). All other statistical calculations were performed using SPSS Statistics 28.0.0 (IBM Corporation, Armonk, New York, USA).

## Results

### Immunogenic LOR to infliximab or adalimumab in IBD patients receiving therapeutic drug monitoring

**Fig. 1:**
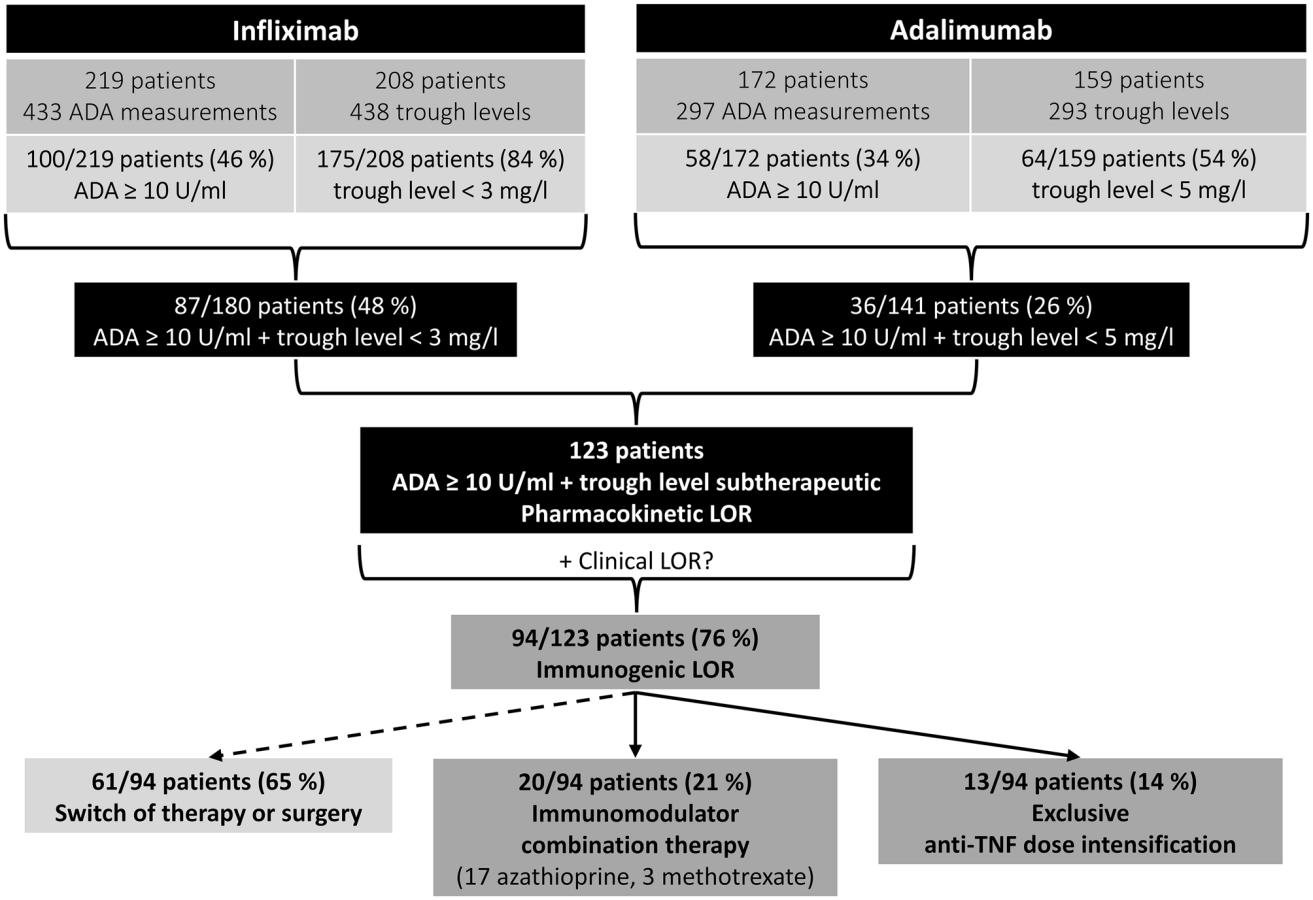
Flow chart depicting the criteria for selecting inflammatory bowel disease patients. ADA, anti-drug antibodies; LOR, loss of response

Between January 1, 2013 and December 31, 2020, 438 IFX trough level measurements in 208 IBD patients and 293 ADL trough level measurements in 159 IBD patients were identified. Regarding IFX-ADA, 433 measurements were performed in 219 IBD patients, and 100 of them (46%) displayed elevated IFX-ADA levels. Furthermore, of 172 IBD patients with 297 ADA-ADL measurements, only 58 (34%) exhibited elevated ADL-ADA levels (*P* = 0.017; Fig. 1).

Focusing on the patients in whom trough levels and ADA levels were measured simultaneously, a subtherapeutic trough level along with elevated ADA (pharmacokinetic LOR) was identified in 87 out of 180 patients (48%) treated with IFX and 36 out of 141 patients (26%) treated with ADL (*P* = 3.1 × 10^−5^; Fig. 1). These 123 IBD patients (87 IFX-treated and 36 ADL-treated) with a pharmacokinetic LOR were further screened for the presence of clinical LOR. Their demographic and clinical characteristics are presented in Table 1. Clinical LOR was identified in 94 of these patients (76%; 65 IFX-treated, 29 ADL-treated). Altogether, therapeutic drug monitoring (TDM) with concomitant ADA and trough level measurements detected ADA-mediated clinical LOR more frequently in IFX-treated (65/180, 36%) than in ADL-treated (29/141, 21%; *P* = 2.9 × 10^−3^) patients with IBD.

**Table 1:** Demographic and clinical characteristics of the study population. Inflammatory bowel disease patients with neutralizing anti-drug antibodies and subtherapeutic trough levels are divided into patients receiving infliximab (left) and patients receiving adalimumab (right)

**Characteristics**	**IBD patients with pharmacokinetic LOR** **(*n*** **= 123)**	
	**Infliximab** (*n* = 87)	**Adalimumab** (*n* = 36)
**Demographics**		
- Age, yrs (median [Q1, Q3]) - Females (*n* [%]) - BMI, kg/m^2^ (median [Q1, Q3])	45 (38, 54) 6 (46) 26 (23, 31)	41 (32, 52) 20 (56) 29 (24, 33)
**Smokers** (*n* [%])	20 (23)	9 (25)
**Prior anti-TNF therapy** (*n* [%])	32 (37)	15 (42)
**Prior immunomodulator therapy** (*n* [%])	55 (63)	27 (75)
**Disease duration**, yrs (median [Q1, Q3])	10 (6, 17)	9 (5, 15)
**Age at diagnosis**, yrs (median [Q1, Q3])	30 (27, 33)	31 (24, 40)
**Disease type**		
- Crohn’s disease - Ulcerative colitis	61 (70) 26 (30)	27 (75) 9 (25)
**Crohn’s disease location (among respective patients)**		
- Ileum isolated (*n* [%]) - Colon isolated (*n* [%]) - Ileocolic (*n* [%]) - Ileocolic + jejunum (*n* [%]) - Ileocolic + upper gastrointestinal tract (*n* [%])	3 (5) 14 (23) 30 (49) 4 (7) 10 (16)	1 (4) 5 (19) 13 (48) 1 (4) 7 (26)
**Extent of ulcerative colitis (among respective patients)**		
- Proctitis (*n* [%]) - Left-sided colitis (*n* [%]) - Pancolitis (*n* [%])	4 (15) 9 (35) 13 (50)	1 (11) 4 (44) 4 (44)
**Complications**		
- Fistula (*n* [%]) - Stenosis (*n* [%]) - Abscess (*n* [%]) - Extraintestinal manifestations (*n* [%]) - Perianal Crohn’s disease (*n* [%])	31 (36) 25 (29) 23 (26) 28 (32) 24 (28)	16 (44) 15 (42) 10 (28) 17 (47) 11 (31)
**Previous surgery** (*n* [%])	44 (51)	20 (56)

*ADA* anti-drug antibodies, *BMI* body mass index, *IBD* inflammatory bowel disease,* n* number, *Q1* lower (first) quartile, *Q3* upper (third) quartile, *yrs* years

### Immunomodulator comedication is crucial to overcome immunogenic LOR to anti-TNF therapy in IBD

We observed that 61 of the 94 IBD patients with ADA-mediated clinical LOR (65%) switched to another treatment or underwent surgery and therefore did not qualify for this analysis. For the remaining 33 patients (35%), anti-TNF medication was optimized in an attempt to overcome the immunogenic LOR (Fig. 1). In 20 of these 33 IBD patients (61%; immunomodulator combination therapy group), IFX or ADL therapy was continued in combination with azathioprine (17 patients) or methotrexate (3 patients). In the remaining 13 patients (39%; anti-TNF dose intensification group), only the dose of IFX or ADL was intensified, without the addition of an immunomodulator. The demographic and clinical characteristics of IBD patients who received the optimized anti-TNF therapy are shown in Table 2.

**Table 2:** Demographic and clinical characteristics of the inflammatory bowel disease population with an immunogenic loss of response to infliximab or adalimumab receiving the optimized anti-TNF-therapy, which included either a combination therapy with an immunomodulator or a dose intensification of the anti-TNF agent exclusively

**Characteristic**	**IBD patients with optimized anti-TNF therapy after immunogenic LOR(*n*** **= 33)**	
	**Immunomodulator combination therapy** **(azathioprine/methotrexate)** (*n* = 20)	**Exclusive anti-TNF** **dose intensification** **(infliximab/adalimumab)** (*n* = 13)
**Demographics**		
- Age, yrs (median [Q1, Q3]) - Females (n [%]) - BMI, kg/m^2^ (median [Q1, Q3])	41 (37, 52) 10 (50) 27 (22, 32)	45 (38, 54) 6 (46) 26 (23, 31)
**Smokers** (*n* [%])	6 (30)	3 (23)
**Anti-TNF agent**		
- Infliximab (*n* [%]) - Adalimumab (*n* [%])	16 (80) 4 (20)	6 (46) 7 (54)
**Immunomodulator**		
- Azathioprine (*n* [%]) - Methotrexate (*n* [%])	17 (85) 3 (15)	n/a n/a
**Prior anti-TNF therapy** [*n* (%)]	9 (45)	4 (31)
**Duration of current anti-TNF therapy until LOR**, months (median [Q1, Q3])	9 (6, 18)	9 (5, 35)
**Preexisting immunomodulator combination therapy** (*n* [%])	6 (30)	n/a
**Disease type**		
- Crohn’s disease (*n* [%]) - Ulcerative colitis (*n* [%])	14 (70) 6 (30)	10 (77) 3 (23)
**Disease duration**, yrs (median [Q1, Q3])	9 (5, 15)	10 (6, 17)
**Age at diagnosis**, yrs (median [Q1, Q3])	31 (24, 40)	30 (27, 33)
**Disease activity (among respective patients)**		
- Crohn’s disease, HBI (median [Q1, Q3]) - Ulcerative colitis, PMS (median [Q1, Q3])	6 (5, 8) 5 (4, 6)	8 (4, 12) 3 (3, 5)
**Crohn’s disease location (among respective patients)**		
- Ileum isolated (*n* [%]) - Colon isolated (*n* [%]) - Ileocolic (*n* [%]) - Ileocolic + jejunum (*n* [%]) - Ileocolic + upper gastrointestinal tract (*n* [%])	1 (7) 2 (14) 4 (29) 3 (21) 4 (29)	0 (0) 3 (30) 6 (60) 1 (10) 0 (0)
**Extent of ulcerative colitis (among respective patients)**		
- Proctitis (*n* [%]) - Left-sided colitis (*n* [%]) - Pancolitis (*n* [%])	1 (17) 3 (50) 2 (33)	1 (33) 1 (33) 1 (33)
**Complications**		
- Fistula (*n* [%]) - Stenosis (*n* [%]) - Abscess (*n* [%]) - Extraintestinal manifestations (*n* [%]) - Perianal Crohn’s disease (*n* [%])	7 (35) 5 (25) 5 (25) 5 (25) 5 (25)	7 (54) 4 (31) 5 (38) 7 (54) 5 (38)
**Previous surgery** (*n* [%])	11 (55)	8 (62)

*BMI* body mass index, *HBI* Harvey-Bradshaw index, *IBD* inflammatory bowel disease, *LOR* loss of response, *n* number, *n/a* not applicable, *PMS* partial Mayo score, *Q1* lower (first) quartile, *Q3* upper (third) quartile, *yrs* years

In these 33 IBD patients, immunomodulator combination therapy was identified as the crucial factor that significantly influenced the regain of clinical remission [OR (95% CI) 23.00 (4.46–171.14); *P* = 7.3 × 10^−5^], ADA clearance [OR 4.71 (1.16–21.98); *P* = 0.030], and regain of clinical remission along with the pharmacokinetic response [OR 5.57 (1.23–34.43); *P* = 0.025] in univariable logistic regression analyses (Table 3). Additionally, univariable analyses showed that a lower CRP level at the time of LOR is positively associated with ADA clearance [OR 0.93 (0.86–0.99); *P* = 7.7 × 10^−3^], and UC diagnosis is a positive predictor for regain of clinical remission [OR 6.65 (1.21, 69.46); *P* = 0.028]. The key effect of immunomodulator comedication on regain of clinical remission, ADA clearance, and regain of clinical remission along with the pharmacokinetic response was also observed in the multivariable models (Table 3) when adjusted for the potential confounding factors, including age and sex (model 1), BMI (model 2), smoking status (model 3), TNF inhibitor trough levels at the time of LOR (model 4), CRP levels at the time of LOR (model 5), ADA levels at the time of LOR (model 6),as well as UC diagnosis (model 7). Higher TNF inhibitor trough levels at the time of LOR [model 4; OR 1.92 (1.05, 4.18); *P* = 0.034] and lower CRP levels at the time of LOR [model 5; OR 0.95 (0.86, 0.99); *P* = 8.4 × 10^−3^] favored ADA clearance by immunomodulator combination therapy in multivariable models. Diagnosis of UC remained a positive predictor for regain of clinical remission in multivariable analysis [model 7; OR 56.75 (2.69, 11,672.48); *P* = 5.7 × 10^−3^] and in additional multivariable models adjusted for sex, age, BMI, smoking status, TNF inhibitor trough levels at the time of LOR, CRP levels at the time of LOR, and ADA levels at the time of LOR (Supplementary Table 1, Online Resource 1).

**Table 3:** Univariable and multivariable Firth’s logistic regression models analyzing the influence of immunomodulator combination therapy, anti-TNF dose intensification, and several potential demographic and clinical confounding factors on regain of clinical remission, clearance of anti-drug antibodies, and regain of clinical remission along with the pharmacokinetic response in 33 inflammatory bowel disease patients receiving optimized anti-TNF therapy after immunogenic loss of response

**Variables**	**Regain of clinical remission**		**Clearance of anti-drug antibodies**		**Regain of clinical remission and pharmacokinetic response**	
	**(Adjusted) OR (95% CI)**	***P*-value**	**(Adjusted) OR (95% CI)**	***P*-value**	**(Adjusted) OR (95% CI)**	***P*-value**
**Univariable models**						
Immunomodulator combination therapy^a^	23.00 (4.46, 171.14)	7.3 × 10^−5^	4.71 (1.16, 21.98)	0.030	5.57 (1.23, 34.43)	0.025
Anti-TNF dose intensification^b^	0.94 (0.14, 5.64)	0.95	1.85 (0.31, 12.63)	0.50	0.92 (0.15, 6.28)	0.93
Male (vs. female)	0.69 (0.17, 2.65)	0.59	0.55 (0.14, 2.11)	0.39	0.44 (0.10, 1.72)	0.24
Age, yrs	1.00 (0.93, 1.06)	0.90	1.01 (0.95, 1.08)	0.77	1.01 (0.94, 1.07)	0.87
BMI, kg/m^2^	1.02 (0.90, 1.17)	0.74	1.06 (0.93, 1.21)	0.40	1.06 (0.93, 1.21)	0.41
Active smoking^c^	0.50 (0.11, 2.21)	0.36	1.04 (0.24, 4.75)	0.96	0.74 (0.15, 3.32)	0.70
TNF inhibitor trough level T1, mg/L	0.89 (0.52, 1.51)	0.66	1.44 (0.85, 2.80)	0.19	1.02 (0.59, 1.72)	0.94
CRP, mg/L	0.99 (0.95, 1.01)	0.28	0.93 (0.86, 0.99)	7.7 × 10^−3^	0.96 (0.88, 1.00)	0.072
ADA level T1, U/mL	1.00 (0.99, 1.00)	0.54	1.00 (0.99, 1.00)	0.31	1.00 (0.99, 1.00)	0.23
Infliximab (vs. adalimumab)	2.02 (0.49, 8.66)	0.33	1.01 (0.24, 4.13)	0.99	0.69 (0.17, 2.92)	0.61
Ulcerative colitis (vs. Crohn’s disease)	6.65 (1.21, 69.46)	0.028	1.86 (0.42, 9.36)	0.42	1.33 (0.29, 5.98)	0.70
**Multivariable model 1**						
Immunomodulator combination therapy^a^	19.07 (3.91, 135.45)	1.2 × 10^−4^	4.54 (1.13, 21.20)	0.033	5.50 (1.21, 35.13)	0.026
Male [ref. female]	0.62 (0.10, 3.57)	0.59	0.56 (0.13, 2.31)	0.43	0.43 (0.09, 1.81)	0.25
Age, yrs	1.01 (0.93, 1.10)	0.85	1.01 (0.95, 1.09)	0.68	1.01 (0.94, 1.08)	0.76
**Multivariable model 2**						
Immunomodulator combination therapy^a^	18.72 (3.87, 130.34)	1.3 × 10^−4^	4.43 (1.10, 20.45)	0.035	5.18 (1.17, 31.45)	0.029
BMI, kg/m^2^	1.01 (0.85, 1.20)	0.95	1.05 (0.92, 1.21)	0.50	1.04 (0.91, 1.20)	0.52
Age, yrs	1.01 (0.93, 1.10)	0.80	1.01 (0.95, 1.09)	0.67	1.01 (0.94, 1.08)	0.78
**Multivariable model 3**						
Immunomodulator combination therapy^a^	30.74 (5.02, 382.02)	4.8 × 10^–5^	4.52 (1.14, 20.88)	0.032	5.60 (1.25, 35.20)	0.023
Active smoking^c^	0.19 (0.02, 1.43)	0.11	0.92 (0.19, 4.52)	0.91	0.61 (0.11, 2.97)	0.55
Age, yrs	1.01 (0.93, 1.10)	0.80	1.02 (0.95, 1.09)	0.62	1.01 (0.95, 1.09)	0.71
**Multivariable model 4**						
Immunomodulator combination therapy^a^	24.23 (4.33, 260.94)	9.8 × 10^–5^	9.24 (1.77, 80.71)	6.7 × 10^−3^	6.49 (1.32, 52.67)	0.020
TNF inhibitor trough level T1, mg/L	1.30 (0.65, 2.71)	0.45	1.92 (1.05, 4.18)	0.034	1.29 (0.69, 2.48)	0.42
Age, yrs	1.02 (0.93, 1.11)	0.73	1.03 (0.96, 1.12)	0.40	1.02 (0.95, 1.10)	0.61
**Multivariable model 5**						
Immunomodulator combination therapy^a^	25.95 (4.80, 213.72)	5.2 × 10^−5^	5.64 (1.22, 30.54)	0.027	5.96 (1.28, 37.67)	0.022
CRP, mg/L	0.98 (0.94, 1.00)	0.094	0.95 (0.86, 0.99)	8.4 × 10^−3^	0.97 (0.89, 1.00)	0.066
Age, yrs	0.99 (0.89, 1.09)	0.87	1.00 (0.91, 1.08)	0.93	1.00 (0.93, 1.08)	1.00
**Multivariable model 6**						
Immunomodulator combination therapy^a^	28.89 (4.91, 340.56)	5.0 × 10^−5^	5.73 (1.32, 31.62)	0.019	7.10 (1.46, 51.24)	0.014
ADA level T1, U/mL	0.99 (0.99, 1.00)	0.15	1.00 (0.99, 1.00)	0.15	0.99 (0.99, 1.00)	0.11
Age, yrs	1.02 (0.94, 1.12)	0.63	1.02 (0.96, 1.10)	0.50	1.02 (0.95, 1.11)	0.50
**Multivariable model 7**						
Immunomodulator combination therapy^a^	64.33 (6.20,9109.96)	4.4 × 10^−5^	4.36 (1.09, 20.00)	0.037	5.24 (1.19, 31.72)	0.028
Ulcerative colitis (vs. Crohn’s disease)	56.75 (2.69, 11,672.48)	5.7 × 10^−3^	1.61 (0.29, 9.91)	0.58	1.07 (0.19, 5.89)	0.94
Age, yrs	0.96 (0.85, 1.05)	0.37	1.01 (0.94, 1.09)	0.81	1.01 (0.94, 1.09)	0.74

*ADA* anti-drug antibodies, *BMI* body mass index, *CI* confidence interval, *CRP* C-reactive protein *IBD *inflammatory bowel disease,  *OR* odds ratio, *T1* time point 1 (time of loss of response), *vs.* versus, *yrs* years

^a^versus exclusive anti-TNF dose intensification

^b^versus no anti-TNF dose intensification

^c^versus not smoking

**Fig. 2:**
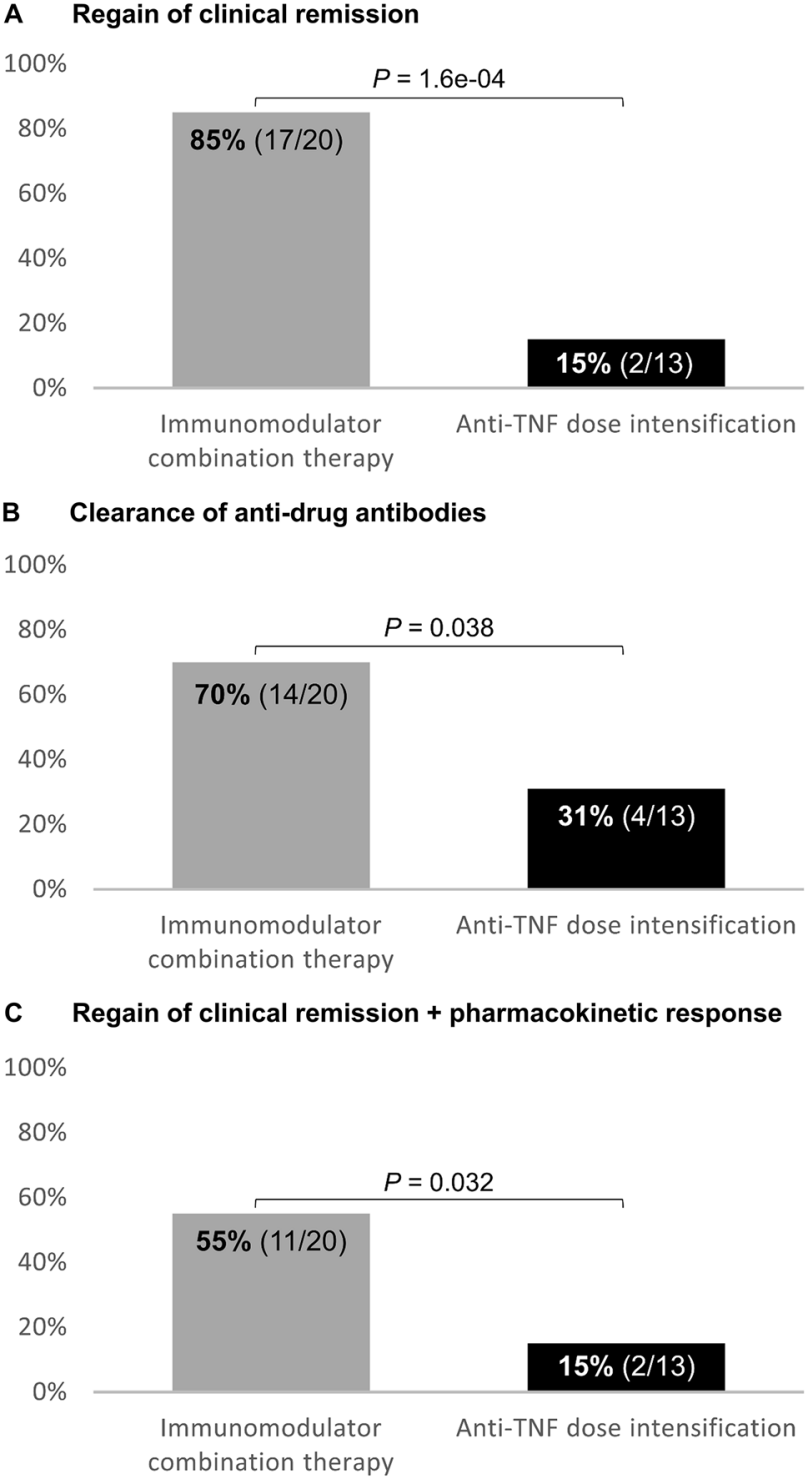
Regain of clinical remission (**A**), clearance of anti-drug antibodies (**B**), and regain of clinical remission along with the pharmacokinetic response (**C**) in inflammatory bowel disease patients receiving immunomodulator combination therapy (left bar, gray) or anti-TNF dose intensification (right bar, black) following the immunogenic loss of response to anti-TNF therapy. Results were compared using the Fisher’s exact test. ADA, anti-drug antibodies

In the immunomodulator combination therapy group, 17 out of 20 patients (85%) regained clinical remission after a median duration of 9 (6, 16) months, in contrast to only 2 out of 13 patients (15%) after 8 and 20 months, respectively, in the anti-TNF dose intensification group (*P* = 1.6 × 10^−4^, Fig. 2A). ADA was completely eradicated in 14 patients (70%) of the immunomodulator comedication therapy group after a median duration of 8 (5, 15) months and only in 4 patients (31%) of the anti-TNF dose intensification group after a median duration of 2 (1, 8) months (*P* = 0.038, Fig. 2B). Regarding the combined endpoint of pharmacokinetic response and clinical remission, 11 patients (55%) of the immunomodulator combination therapy group exhibited therapeutic trough levels ≥ 3 mg/L for IFX and ≥ 5 mg/L for ADL along with no ADA (< 10 U/mL) and clinical remission after a median duration of 12 (6, 24) months. However, in the anti-TNF dose intensification group, regain of clinical remission along with the pharmacokinetic response was observed only in two patients (15%) after 8 and 20 months, respectively (*P* = 0.032, Fig. 2C). Importantly, no differences were observed in the median ADA levels at the time of LOR (time point T1) between the immunomodulator combination therapy group [46.10 U/mL (15.18, 142.00)] and the anti-TNF dose intensification group [42.70 U/mL (15.40, 142.00); *P* = 0.99] and in median T1 trough levels between the two groups [1.00 mg/L (0.43, 2.25) vs. 1.60 mg/L (0.45, 3.21), *P* = 0.40].

In the combination therapy group, the second measurement of ADA and trough levels after a median duration of 8 (6, 15) months was available for all 20 patients, since all patients remained on immunomodulator comedication throughout the period of study. In the anti-TNF dose intensification group, the second measurement of ADA and trough levels after a median duration of 6 (2, 8) months was available for 8 out of 13 patients (62%). Dose intensification was stopped in the remaining five patients (38%) after a median duration of 4 (2, 6) weeks due to the disease flare that necessitated treatment switching.

Excluding 5 patients with no second measurement, 17 out of 20 patients (85%) in the immunomodulator combination therapy group and 2 out of 8 patients (25%) in the anti-TNF dose intensification group regained clinical remission (*P* = 4.8 × 10^−3^). Clinical remission following dose intensification was accompanied by ADA clearance and a pharmacokinetic response in both cases. The two responders to dose intensification had particularly low ADA titers (14.62 U/mL and 20.50 U/mL).

### Successful clearance of ADA following immunomodulator comedication

**Fig. 3:**
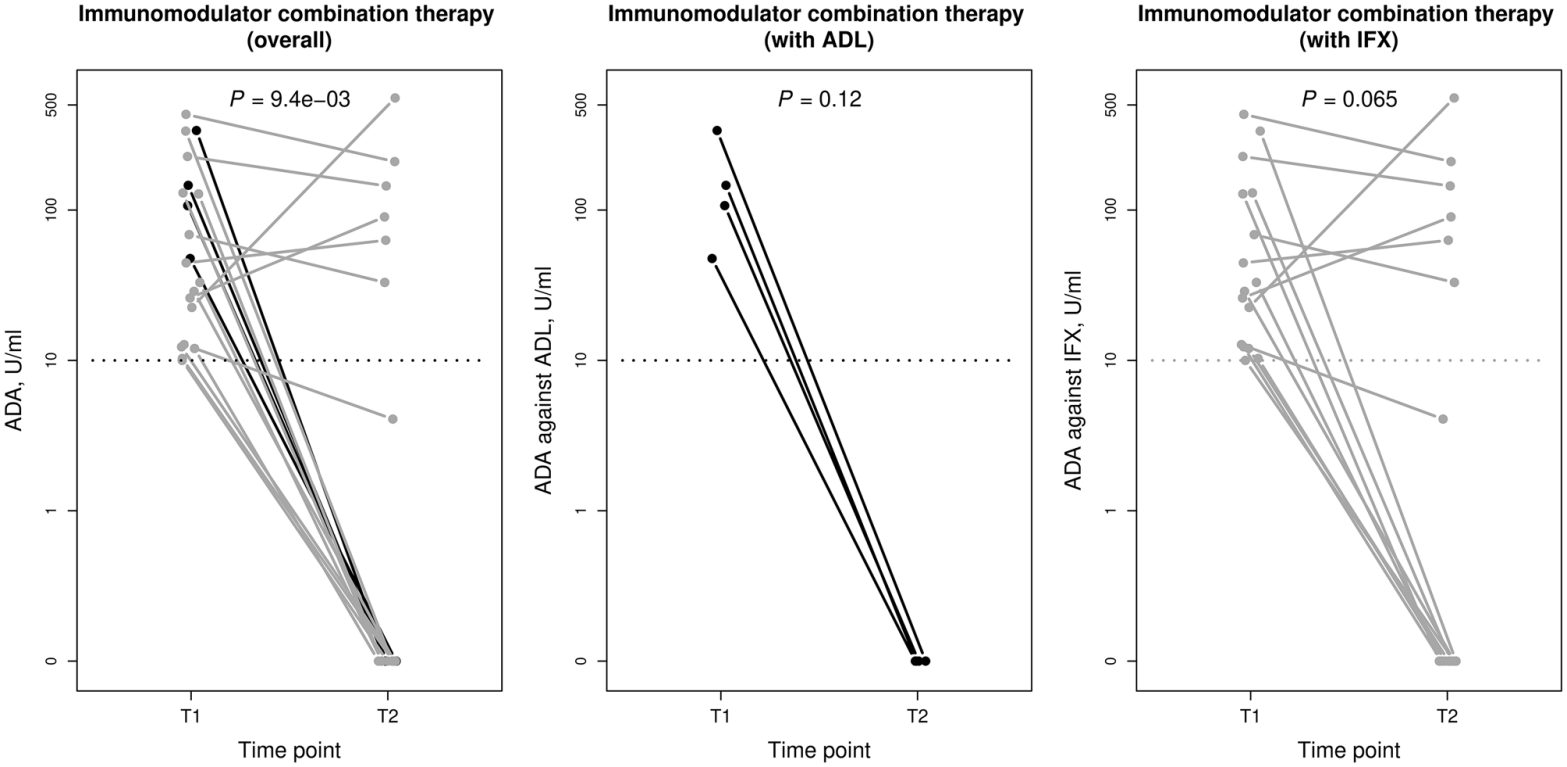
Levels of anti-adalimumab (black) and anti-infliximab (gray) antibodies in inflammatory bowel disease patients receiving immunomodulator combination therapy following immunogenic loss of response. Cut-off value of 10 U/mL for positive anti-drug antibodies is marked as a horizontal line. Data were compared using the Wilcoxon signed rank test. ADA, anti-drug antibodies; ADL, adalimumab; IFX, infliximab; T1, time point 1 (time of immunogenic loss of response); T2, time point 2

**Fig. 4:**
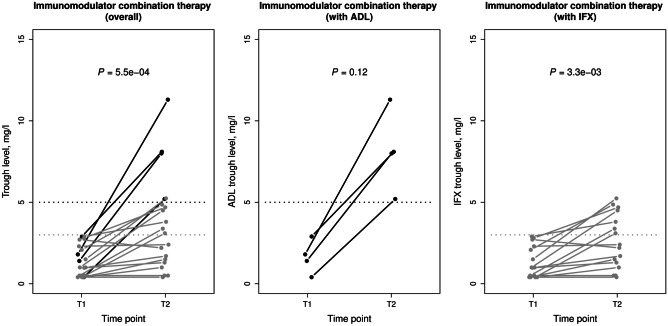
Adalimumab (black) and infliximab (gray) trough levels in inflammatory bowel disease patients receiving immunomodulator combination therapy following immunogenic loss of response. Cut-off values of 5 mg/L for therapeutic ADL trough levels and of 3 mg/L for therapeutic IFX trough levels are marked as horizontal lines. Data were compared using the Wilcoxon signed rank test. ADL, adalimumab; IFX, infliximab; T1, time point 1 (time of immunogenic loss of response); T2, time point 2

In the immunomodulator combination therapy group, median ADA levels decreased significantly from 46.05 (15.18, 142.00) U/mL at time point T1 to 0.00 (0.00, 55.46) U/mL at time point T2 (*P* = 9.4 × 10^−3^; Fig. 3). Immunomodulator comedication led to a complete eradication of ADA (< 10 U/mL) in all 4 ADL-treated patients (100%; 2 patients on 2 mg/kg azathioprine and 2 on 1 mg/kg azathioprine) and in 10 out of 16 IFX-treated patients (63%; 5 patients on 1 mg/kg azathioprine, 3 on methotrexate, and 2 on 2 mg/kg azathioprine;* P* = 0.27 compared to ADL-treated). In 6 of the 16 IFX-treated patients (38%; 3 patients on 1 mg/kg azathioprine and 3 on 2 mg/kg azathioprine), ADA against IFX could not be eradicated. All patients with a complete clearance of ADA regained clinical remission. Moreover, in all four ADL-treated patients, therapeutic trough levels (> 5 mg/L) were fully restored (Fig. 4; *P* = 0.12). Regarding IFX-treatment, 7 of the 10 patients (70%) with complete ADA eradication regained therapeutic IFX levels (> 3 mg/L) during the time of evaluation while the remaining 3 (30%) still had subtherapeutic IFX trough levels (1.70, 2.20, and 2.40 mg/L) at time point T2. Nevertheless, all IFX-treated patients experienced a regain of clinical remission. In summary, in the immunomodulator combination therapy group, median anti-TNF trough levels increased significantly from 1.00 (0.43, 2.25) U/mL at the time point T1 to 3.24 (1.35, 5.12) U/mL at time point T2 (Fig. 4; *P* = 5.5 × 10^–4^). Results of patients with no immunomodulator comedication are provided in Supplementary Fig. 1 (Online Resource 2).

### Higher TNF inhibitor trough levels at the time of LOR can predict ADA clearance and regain of clinical remission in immunomodulator combination therapy group

**Fig. 5:**
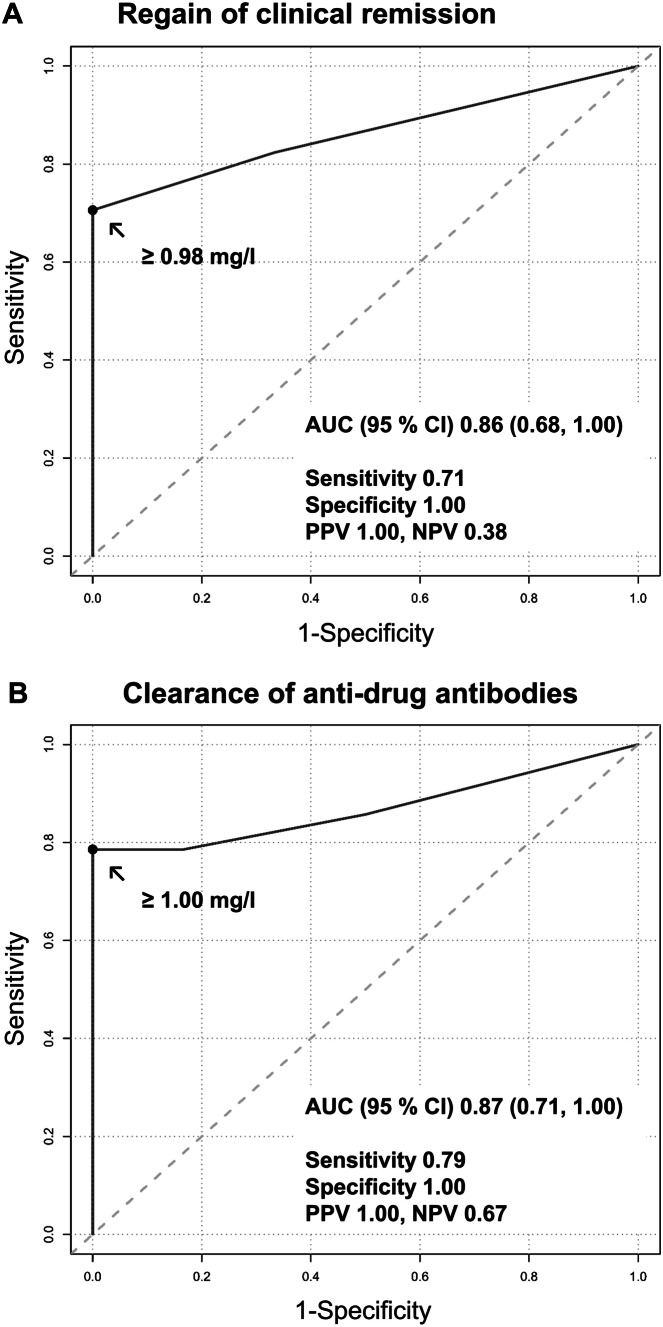
Receiver operating characteristics curves showing the performance of TNF inhibitor trough levels at time point T1 (immunogenic loss of response) for predicting the regain of clinical remission (**A**) and the clearance of anti-drug antibodies (**B**) in inflammatory bowel disease patients receiving immunomodulator combination therapy after immunogenic loss of response. AUC, area under the curve; CI, confidence interval; NPV, negative predictive value; PPV, positive predictive value

Based on the results of multivariable regression analysis (Table 3), we performed the ROC curve analysis to evaluate the performance of TNF inhibitor trough levels and CRP levels at time point T1 for predicting the endpoints (i) regain of clinical remission, (ii) ADA clearance, and (iii) regain of clinical remission along with the pharmacokinetic response in the immunomodulator combination therapy group. Trough levels ≥ 0.98 mg/L and ≥ 1.00 mg/L at time point T1 successfully predicted the regain of clinical remission [AUC (95% CI) 0.86 (0.68, 1.00), sensitivity 0.71, specificity 1.00; Fig. 5A] and ADA clearance [AUC (95% CI) 0.87 (0.71, 1.00), sensitivity 0.79, specificity 1.00; Fig. 5B], respectively, in the immunomodulator combination therapy group with a positive predictive value (PPV) of 100%. Patients under immunomodulator comedication who exhibited a complete eradication of ADA displayed significantly higher T1 trough levels [median 1.65 (0.88, 2.73) U/mL] than those without complete ADA eradication [0.45 (0.40, 0.62) U/mL; *P* = 7.9 × 10^−3^]. CRP levels < 38.40 mg/L at time point T1 favored ADA clearance [AUC (95% CI) 0.80 (0.56, 1.00), sensitivity 1.00, specificity 0.50] following immunomodulator combination therapy with a positive predictive value of 82%. Patients under immunomodulator comedication showing a complete eradication of ADA had significantly lower T1 CRP levels [6.80 (2.00, 15.50) mg/L] than those without complete eradication [26.15 (8.00, 99.60) mg/L; *P* = 0.041]. Results of the ROC curve analysis are shown in Supplementary Table 2 (Online Resource 3). Furthermore, there was no detectable influence of T1 absolute ADA levels on the endpoints (i), (ii), and (iii) in the multivariable regression analysis (Table 3).

### Elevated systemic CRP levels at the time of LOR can predict the failure of TNF inhibitor dose intensification

In the univariable logistic regression analysis (Table 3), lower systemic CRP levels at the time of immunogenic LOR favored ADA clearance. In contrast, no association was observed between TNF inhibitor dose intensification and ADA clearance. Moreover, IBD patients who exclusively received TNF inhibitor dose intensification as a strategy to overcome immunogenic LOR were less likely to achieve the three endpoints compared to those who received immunomodulator combination therapy (Fig. 2). We further tested the diagnostic performance of higher CRP levels at time point T1 for predicting the failure of TNF inhibitor dose intensification. Elevated systemic CRP levels at time point T1 (≥ 6.00 mg/L) were significantly associated with the failure of TNF inhibitor dose intensification with respect to regain of clinical remission [AUC (95% CI) 0.89 (0.68, 1.00), sensitivity 0.82, specificity 1.00, PPV 1.00], ADA clearance [AUC (95% CI) 0.81 (0.56, 1.00), sensitivity 0.89, specificity 0.75, PPV 0.89], and regain of clinical remission along with the pharmacokinetic response [AUC (95% CI) 0.89 (0.68, 1.00), sensitivity 0.82, specificity 1.00, PPV 1.00]. Complete ROC data including the diagnostic performance of higher ADA levels and lower TNF inhibitor trough levels at time point T1 for determining the failure of TNF inhibitor dose intensification are given in Supplementary Table 2 (Online Resource 3). ADA levels ≥ 38.60 U/mL and ≥ 57.36 U/mL were associated with a nominal PPV of 100% for no possible regain of clinical remission [AUC (95% CI) 0.77 (0.46, 1.00), sensitivity 0.73, specificity 1.00] and for no possible ADA clearance [AUC (95% CI) 0.75 (0.47, 1.00), sensitivity 0.67, specificity 1.00], respectively, albeit with no diagnostic validity. Combining ADA levels ≥ 57.36 U/mL with T1 trough levels < 2.01 mg/L to predict no ADA clearance improved the sensitivity to 0.89 (specificity 0.75, PPV 0.89; Supplementary Table 3, Online Resource 3). Detailed outcome data of anti-TNF dose intensification attempts separately for patients on ADL and IFX, and separately for UC and CD patients are provided as supplementary material (Supplementary Text, Online Resource 4).

### Immunomodulator comedication reverses immunogenic LOR to anti-TNF therapy in patients with CD and UC

We found that 14 out of 24 CD patients (3 on ADL and 11 on IFX) underwent immunomodulator combination therapy following immunogenic LOR, and among them, 11 (79%) responded to immunomodulator comedication with clinical remission. A complete clearance of ADA was detected in 10 of the 14 CD patients (71%) after a median duration of 11 (7, 20) months. Furthermore, 9 of the 14 patients (64%) regained clinical remission and a full pharmacokinetic response with therapeutic trough levels and no ADA after a median duration of 12 (7, 25) months.

Regarding UC, six out of nine patients with immunogenic LOR (one on ADL, five on IFX) underwent immunomodulator combination therapy. All six patients (100%) regained clinical remission with immunomodulator combination therapy. Complete clearance of ADA was observed in four of the six UC patients (67%) after a median duration of 4 (2, 5) months. However, therapeutic trough levels were not achieved in two of these four patients until the end of the observation period. Furthermore, two of these six patients (33%) regained clinical remission along with a full pharmacokinetic response, therapeutic trough levels, and no ADA after 5 and 13 months, respectively.

Of note, complete eradication of ADA was achieved significantly earlier [4 (3, 5) months, exact median 125 (89, 141) days] in UC patients than in CD patients [ 11 (7, 19) months, exact median 323 (211, 568) days; *P* = 0.0058].

Concerning side effects, immunomodulator combination therapy had to be stopped in one female CD patient due to an allergic infusion reaction to IFX with facial edema shortly after introducing the comedication with azathioprine. Another female CD patient receiving a combination of IFX and methotrexate experienced bacterial tonsillitis, which was treated with antibiotics. A self-limiting elevation of alanine aminotransferase to more than twice the upper limit of normal was recorded in the same patient. A third male UC patient treated with azathioprine and IFX developed an elevation of gamma-glutamyl transferase to more than three times the upper limit of normal, which was also self-limiting. In the two latter cases, combination therapy could be continued without adjustments. Altogether, protocolled side effects occurred in 3 out of 20 patients (15%) receiving immunomodulator comedication. Side effects were recorded in a comparable proportion of 2 out of 13 patients (15%; *P* = 1.00) in the anti-TNF dose intensification group: One female UC patient had to stop ADL therapy because of a lupus-like syndrome, one male CD patient could continue intensified IFX medication despite mild anti-TNF-induced psoriasis that was treated with topical steroids.

### Dosages of immunomodulators and anti-TNF agents

In 6 out of 20 patients (30%) of the immunomodulator combination therapy group, the preexisting immunomodulator combination therapy with azathioprine (5 patients) or methotrexate (1 patient) was continued following the immunogenic LOR (Table 1). In remaining 14 patients (70%), combination therapy with azathioprine (12 patients) or methotrexate (2 patients) was newly introduced. Furthermore, 10 of the 20 patients (50%) received a therapeutic dose of azathioprine (7 patients, 2 mg/kg) or methotrexate (3 patients, 15 mg methotrexate/week after an initial dose of 25 mg/week subcutaneously for 8 weeks), while the remaining 10 patients (50%) were started on a low-dose azathioprine regimen (1 mg/kg). In 3 of the 16 IFX-treated patients (19%), IFX dose was increased in addition to immunomodulator comedication; in all three patients, the interval was shortened from 8 to 6 weeks, and in two patients, the dose was increased from 4 to 9 mg/kg and from 5 to 7 mg/kg, respectively. In three of the four patients (75%) on ADL who received immunomodulator comedication, ADL dose was escalated from 40 mg every 2 weeks to 40 mg every week.

In all seven ADL-treated patients of the dose intensification group, the dosage interval was reduced from 40 mg every two weeks to 40 mg every week. In four out of six IFX-treated patients, the dosage interval (dose 5 mg/kg) was shortened from 8 to 4 weeks (two patients), from 8 to 6 weeks (one patient), or from 6 to 4 weeks (one patient). In one patient, the IFX dose was increased from 400 to 500 mg (6 mg/kg) every 8 weeks. A combined approach of dose escalation from 6 to 10 mg/kg and interval shortening from 8 to 6 weeks was tried in one further patient.

## Discussion

Immunogenic LOR due to the formation of neutralizing ADA against TNF inhibitors is one of the most common causes of therapy failure in IBD patients [6, 29, 30]. In this real-world retrospective analysis of IBD patients who received TDM at a tertiary care center, we aimed to investigate whether immunomodulator comedication can reverse immunogenic clinical LOR in patients with IBD.

Our results showed that immunogenic clinical LOR was more frequent in IFX-treated than in ADL-treated patients, which is in accordance with previous studies [7]. Due to its chimeric design, IFX is known to be more immunogenic than the completely humanized ADL [30].

Thirty-three IBD patients who did not undergo a therapy switch after immunogenic LOR and proceeded with an optimized anti-TNF regimen with or without an immunomodulator were included in our analyses. Univariable and multivariable logistic regression analyses revealed that immunomodulator comedication is a crucial factor that promotes the reversal of immunogenic LOR. Under combination therapy with an immunomodulator, IBD patients who adhered to their initial TNF inhibitor were able to regain clinical remission and pharmacokinetic response. More than half of the 20 patients on immunomodulator comedication exhibited a complete eradication of ADA and an increase in trough levels of IFX or ADL to therapeutic values along with clinical remission within a median duration of 12 months. In contrast, dose intensification of the respective TNF inhibitor in the remaining 13 IBD patients was clinically and pharmacokinetically effective only in 2 patients with particularly low ADA levels.

TNF inhibitor dose intensification may lead to clinical improvement and reduction in ADA levels in some patients with immunogenic LOR and is therefore occasionally used in clinical practice [31–36]. However, our data support this strategy only in patients with low systemic CRP levels (< 6.00 mg/L) and marginally elevated ADA levels. Consistent with other studies [37, 38], our study showed that the application of a cut-off value ≥ 38.60 U/mL for ADA levels resulted in a PPV of 100% for the prediction of a clinical non-responsiveness to increased drug dosages. Regarding the failure of ADA clearance, a cut-off value ≥ 57.36 U/mL for ADA levels also resulted in a PPV of 100%. Therefore, a therapeutic switch to a second-line anti-TNF antibody or another biologic is generally recommended for patients with low or undetectable drug levels and high ADA titers [19, 37, 38]. This can be detrimental for IBD patients who are dependent on anti-TNF regimens because of fistulizing CD [3] or a TNF pathway-dominant disease with immunogenic secondary LOR to TNF blockage, especially if the patient is already treated with a second-line anti-TNF and a third-line anti-TNF agent is not an option. The latter is the case in many countries outside the USA, where IFX and ADL are the only two approved anti-TNF agents for CD and certolizumab is not available [2]. Furthermore, although respective head-to-head studies are mostly missing, recent network meta-analyses of phase 2 and phase 3 randomized controlled trials have suggested IFX as the most effective treatment option in UC [5], and IFX and ADL as the two most potent first-line choices in CD therapy [4]. This emphasizes the importance of anti-TNF therapy optimization before switching to a potentially less effective one. Second-line therapies, in class or out of class, generally tend to be less effective in anti-TNF experienced patients compared to anti-TNF naïve patients [12, 20, 23].

Herein, we suggest an effective alternative to switching to another biologic in IBD cases with ADA-mediated immunogenic clinical LOR to IFX or ADL. Furthermore, we confirm the findings of a few existing small studies demonstrating the efficacy of immunomodulator combination therapy to overcome the ADA-mediated clinical LOR in IBD [13–18].

Our study has several strengths. First, it systematically analyzes the real-world data reflecting the patient care in a large tertiary care IBD center and is therefore directly applicable to daily clinical routine. Second, our definition of LOR included a true clinical LOR along with subtherapeutic trough levels and ADA positivity; therefore, the possible overestimation of transient and clinically non-relevant ADA is unlikely. Third, in contrast to previous reports [13–17], our cohort study included a comparison group. IBD patients receiving immunomodulator comedication were compared to patients receiving dose optimization of the anti-TNF agent in order to reverse the immunogenic LOR; some previous studies suggest dose optimization of the existing anti-TNF agent to reduce ADA levels [32–36]. Fourth, the effect of immunomodulator comedication was controlled in multivariable logistic regression analyses by adjusting for potential cofounding factors like CRP levels, BMI, smoking status, and trough levels at the time of LOR as these factors might also affect the clearance of ADA. A high BMI, active smoking, and low trough levels are risk factors for the development of ADA [7]. We found that TNF inhibitor trough levels of about 1 mg/L or above at the time of immunogenic LOR could predict the success of the immunomodulator combination therapy with respect to ADA clearance and regain of clinical remission, respectively, with a PPV of 100%. CRP levels below 38.40 mg/L at the time of LOR also predicted ADA clearance (PPV of 82%), which could be interpreted as a direct consequence of higher TNF inhibitor trough levels. Furthermore, this study is the first that included adult patients not only suffering from CD almost exclusively but also from UC. Our study suggests that immunomodulator combination therapy is an effective strategy to resolve immunogenic LOR to IFX or ADL in adult UC patients. Remarkably, all UC patients on immunomodulator combination therapy regained clinical remission after immunogenic LOR, and ADA clearance could be detected earlier in UC patients than in CD patients. This might be explained by a superior clinical effectiveness of immunomodulator monotherapy in UC in general [39]. Concerning regain of clinical remission after immunogenic LOR, univariable and multivariable logistic regression analyses revealed a potential advantage for UC patients over CD patients. While the exclusive use of drug-sensitive assays that can detect ADA only in the absence or with low concentrations of the respective drug [16, 32] has been a subject of debate in previous studies [16], the drug-tolerant assay can reduce the risk of underestimated ADA titers in the presence of high drug levels and was available for the majority of ADA measurements in the study at hand. Although ADA detection and its clearance could be more reliable, we could not use the data from the drug-tolerant assay exclusively. However, it is important to note that low concentrations of ADA, although not detectable using the drug-sensitive assay, seem clinically non-relevant. Clinically relevant high concentrations of ADA can be detected using the drug-sensitive assay [32].

The two main weaknesses of our study are the retrospective design and the modest sample size of 33 patients with an optimized anti-TNF therapy after the immunogenic LOR. Nevertheless, patients analyzed in our investigation outnumber those analyzed in all but one [16] previous studies [13–15, 17] on reversal of immunogenic LOR to anti-TNF therapy in adult IBD patients with immunomodulator combination therapy. Because of obstacles such as the time lag in obtaining results and laboratory costs, clinicians working outside the university hospitals avoid TDM application, and the lack of TDM data hinders large multicentric retrospective studies [40]. Although the previously published independent small investigations are consistent with our findings [13–18], prospective studies are clinically relevant to confirm the efficient reversal of immunogenic clinical LOR by immunomodulator add-on therapy for IBD. It could be argued, that due to differences in pathophysiology, patients suffering from CD and UC should be separated into two cohorts. However, our modest sample size did not support a separate evaluation. The good response to anti-TNF treatment is one of the common features of both diseases [2, 4, 5]. In this respect, a combined analysis seems justified. Disease-specific differences between CD and UC were included in our analyses.

Our study shows that complete clearance of ADA and restoration of therapeutic drug levels takes time. A complete clearance of ADA and restoration of therapeutic trough levels in clinical and pharmacokinetic responders to immunomodulator combination therapy could be diagnosed after almost 1 year. This is due to the fact that the flexible timing of follow-up drug monitoring was up to the treating physician. Nevertheless, the adherence of all patients to immunomodulator combination therapy over an extended period until the second drug monitoring indeed suggests an earlier clinical response. The clinical response might be due to the synergistic anti-inflammatory effect of the immunomodulator and anti-TNF therapy, which was initially demonstrated for CD in the SONIC trial [41] and for UC in the UC-SUCCESS trial [42]. The pharmacokinetic response could follow later and increased anti-TNF antibody levels may in part be explained by reduced inflammation with lower levels of circulating TNF, which has to be neutralized by anti-TNF antibodies. However, the elimination of ADA can be interpreted immunologically as a direct inhibitory effect of methotrexate and thiopurines on T cell proliferation leading to the attenuation of T cell-dependent antibody production by B cells [43, 44].

Remarkably, half of the patients on immunomodulator comedication in our study were on a half-therapeutic dose regimen of azathioprine (1 mg/kg). It has been suggested that in IBD, low-dose azathioprine is sufficient for a positive effect on IFX therapeutic levels and for the beneficial effects of combination therapy, whereas a higher therapeutic dose is necessary for increasing the clinical efficacy of azathioprine monotherapy [45–47]. Furthermore, low-dose azathioprine comedication also minimizes side effects [47]. However, recent data showed that IFX trough levels are dependent on the dose of combined azathioprine, and azathioprine doses above 1 or 2 mg/kg are associated with significantly higher IFX trough levels [48]. We speculate that a therapeutic dose of azathioprine (2–2.5 mg/kg) would have performed even better in eradicating ADA against IFX in some of our IBD patients. Since half of the IFX-treated patients with complete ADA clearance were on a half-therapeutic azathioprine dose regimen, it seems reasonable to start with a half-therapeutic azathioprine comedication dose in cases of immunogenic LOR to anti-TNF monotherapy and then gradually increase the dose if sufficient ADA clearance is not achieved.

Accumulating evidence regarding the prophylaxis of ADA development [7, 12] and synergistic efficacy [41, 42] supports a general initial combination therapy of IFX with an immunomodulator [49]. However, safety issues have to be taken into account considering the long treatment durations in IBD [49]. Immunomodulator combination therapy significantly increases the risk of lymphoma [50–52] and, almost exclusively in young men, of hepatosplenic T cell lymphoma [53]. In the elderly, risks involve toxicity, infectious complications, and a malignancy risk dependent on the age and treatment duration [52, 54–58]. Therefore, the duration of combination therapy should be as short as possible. For ADL, the DIAMOND trial has casted doubt on the synergistic anti-inflammatory effect of a combination with an immunomodulator [59]. However, evidence from a randomized controlled trial supports a combination therapy with azathioprine for ADA prophylaxis in cases where ADL is the second anti-TNF agent after the immunogenic LOR to IFX and vice versa [12]. The addition of an immunomodulator only in patients with a previous or current immunogenic LOR to the anti-TNF agent or pronounced inflammatory activity could avoid unnecessary double immunosuppression in patients who might never develop ADA and are not likely to benefit from the combination.

In conclusion, combining an immunomodulator with the anti-TNF regimen eradicated ADA and reestablished clinical remission in more than half of the analyzed IBD patients with immunogenic LOR to IFX or ADL. Trough levels of about 1 mg/L or above at the time of LOR can predict the success of this strategy. Furthermore, exclusive dose intensification of the anti-TNF agent alone should not be pursued in IBD patients with elevated systemic CRP and high ADA levels. Limiting the immunomodulator comedication to IBD patients with immunogenic LOR to the anti-TNF monotherapy might be an alternative approach to initial combination therapy for all patients, especially when the risk of malignancy or infection is relevant.

### Supplementary Information

Below is the link to the electronic supplementary material.

Supplementary file1 (PDF 210 KB)

Supplementary file2 (PDF 251 KB)

Supplementary file3 (PDF 223 KB)

Supplementary file4 (PDF 73 KB)

## Data Availability

The datasets generated during and/or analysed during the study are available from the corresponding author on reasonable request.
