# Supplementary material for: Immunomodulator comedication promotes the reversal of anti-drug antibody-mediated loss of response to anti-TNF therapy in inflammatory bowel disease
Source: Int J Colorectal Dis. 2023 Feb 25;38(1):54. doi: 10.1007/s00384-023-04349-1 (PMC9968255; doi:10.1007/s00384-023-04349-1)
Supplement: Supplementary file 1 — Supplementary file1 (PDF 210 KB) [file 384_2023_4349_MOESM1_ESM.pdf]

Online Resource 1

Article: Immunomodulator Comedication Promotes the Reversal of Anti-Drug Antibody-Mediated Loss of Response to Anti-TNF Therapy in Inflammatory Bowel Disease
Journal: International Journal of Colorectal Disease
Authors: Johannes Stallhofer, Jan Guse, Miriam Kesselmeier, Philip Christian Grunert, Kathleen Lange, Robert Stalmann, Verena Eckardt, Andreas Stallmach

Corresponding author: Dr. med. Johannes Stallhofer, Jena University Hospital, Department of Internal Medicine IV, E-mail: johannes.stallhofer@med.uni-jena.de

Supplementary Table 1. Multivariable Firth's logistic regression models analyzing the influence of diagnosis of ulcerative colitis (versus Crohn's disease) and immunomodulator combination therapy on regain of clinical remission, clearance of anti-drug antibodies, and regain of clinical remission along with the pharmacokinetic response in 33 IBD patients receiving optimized anti-TNF therapy after immunogenic loss of response, including sex, age, body mass index, smoking, TNF-inhibitor trough level at time point T1 (time of loss of response), CRP level at time point T1 and ADA level at time point T1 as additional independent covariables.

Variables	Regain of clinical remission		Clearance of anti-drug antibodies		Regain of clinical remission and pharmacokinetic response	
	(adjusted) OR (95% CI)	*P*-value	(adjusted) OR (95% CI)	*P*-value	(adjusted) OR (95% CI)	*P*-value
**Multivariable model 1**						
Immunomodulator combination therapy[a]	51.69 (5.44, 6984.42)	$7.0x10^{-5}$	4.21 (1.05, 19.33)	0.042	5.22 (1.17, 32.53)	0.030
Male [ref. female]	0.55 (0.06, 4.23)	0.55	0.54 (0.12, 2.21)	0.39	0.43 (0.09, 1.79)	0.25
Age, yrs	0.96 (0.85, 1.05)	0.35	1.00 (0.93, 1.08)	0.91	1.01 (0.94, 1.09)	0.85
Ulcerative colitis [vs. Crohn's disease]	42.71 (2.41, 7870.28)	$6.7x10^{-3}$	1.71 (0.32, 10.27)	0.53	1.22 (0.22, 6.91)	0.82
**Multivariable model 2**						
Immunomodulator combination therapy[a]	60.94 (5.83, 8779.77)	$5.4x10^{-5}$	4.09 (1.03, 18.38)	0.045	4.93 (1.13, 29.48)	0.033
BMI, kg/m^2	1.06 (0.88, 1.34)	0.54	1.05 (0.92, 1.21)	0.51	1.04 (0.91, 1.20)	0.54
Age, yrs	0.95 (0.84, 1.05)	0.32	1.01 (0.93, 1.08)	0.88	1.01 (0.94, 1.09)	0.80
Ulcerative colitis [vs. Crohn's disease]	72.37 (2.80, 17854.27)	$5.3x10^{-3}$	1.66 (0.30, 10.60)	0.56	1.04 (0.18, 5.76)	0.96
**Multivariable model 3**						
Immunomodulator combination therapy[a]	64.51 (6.03, 9500.13)	$4.9x10^{-5}$	4.13 (1.04, 18.66)	0.043	5.49 (1.23, 34.54)	0.025
Active smoking[b]	0.46 (0.03, 4.85)	0.52	1.13 (0.20, 6.18)	0.89	0.56 (0.08, 3.22)	0.52
Age, yrs	0.97 (0.86, 1.06)	0.48	1.01 (0.94, 1.09)	0.83	1.02 (0.94, 1.10)	0.66
Ulcerative colitis [vs. Crohn's disease]	37.16 (1.67, 7490.47)	0.019	1.71 (0.27, 11.96)	0.57	0.82 (0.12, 5.30)	0.83
**Multivariable model 4**						
Immunomodulator combination therapy[a]	123.06 (6.64, 157423073.44)	$4.7x10^{-5}$	8.55 (1.66, 72.46)	$8.6x10^{-3}$	6.17 (1.27, 49.04)	0.022
TNF-inhibitor trough level T1, mg/L	1.82 (0.61, 50.85)	0.29	1.90 (1.04, 4.06)	0.035	1.28 (0.69, 2.44)	0.42
Age, yrs	0.97 (0.86, 1.08)	0.53	1.02 (0.94, 1.11)	0.58	1.02 (0.95, 1.10)	0.63
Ulcerative colitis [vs. Crohn's disease]	68.44 (2.62, 4049156.08)	$6.1x10^{-3}$	1.65 (0.28, 11.45)	0.58	1.01 (0.18, 5.49)	0.99
**Multivariable model 5**						
Immunomodulator combination therapy[a]	107.06 (7.67, 28006.78)	$2.2x10^{-5}$	5.30 (1.17, 27.77)	0.030	5.84 (1.27, 36.73)	0.023
CRP, mg/L	0.97 (0.93, 1.01)	0.12	0.95 (0.87, 0.99)	0.011	0.97 (0.89, 1.00)	0.064
Age, yrs	0.92 (0.75, 1.04)	0.20	1.00 (0.91, 1.08)	0.91	1.00 (0.93, 1.08)	0.92
Ulcerative colitis [vs. Crohn's disease]	84.44 (2.71, 50628.40)	$6.8x10^{-3}$	1.03 (0.15, 7.21)	0.98	0.76 (0.12, 4.44)	0.76
**Multivariable model 6**						
Immunomodulator combination therapy[a]	57.85 (5.96, 7815.33)	$4.7x10^{-5}$	5.32 (1.24, 28.77)	0.023	6.96 (1.44, 50.09)	0.014
ADA level T1, U/mL	1.00 (0.99, 1.01)	0.56	1.00 (0.99, 1.00)	0.19	0.99 (0.99, 1.00)	0.11
Age, yrs	0.97 (0.86, 1.08)	0.54	1.02 (0.95, 1.10)	0.61	1.03 (0.95, 1.12)	0.48
Ulcerative colitis [vs. Crohn's disease]	36.66 (1.81, 7656.17)	0.015	1.25 (0.22, 7.75)	0.80	0.81 (0.13, 4.56)	0.81

Abbreviations: ADA, anti-drug antibodies; BMI, body mass index; CI, confidence interval; CRP, C-reactive protein; IBD, inflammatory bowel disease; OR, odds ratio; T1, time point 1 (time of loss of response); vs., versus; yrs, years; [a]versus exclusive anti-TNF dose intensification; [b]versus not smoking.
