# Supplementary material for: Immunomodulator comedication promotes the reversal of anti-drug antibody-mediated loss of response to anti-TNF therapy in inflammatory bowel disease
Source: Int J Colorectal Dis. 2023 Feb 25;38(1):54. doi: 10.1007/s00384-023-04349-1 (PMC9968255; doi:10.1007/s00384-023-04349-1)
Supplement: Supplementary file 2 — Supplementary file2 (PDF 251 KB) [file 384_2023_4349_MOESM2_ESM.pdf]

Online Resource 2

Article: Immunomodulator Comedication Promotes the Reversal of Anti-Drug Antibody-Mediated Loss of Response to Anti-TNF Therapy in Inflammatory Bowel Disease
Journal: International Journal of Colorectal Disease
Authors: Johannes Stallhofer, Jan Guse, Miriam Kesselmeier, Philip Christian Grunert, Kathleen Lange, Robert Stalmann, Verena Eckardt, Andreas Stallmach

Corresponding author: Dr. med. Johannes Stallhofer, Jena University Hospital, Department of Internal Medicine IV, E-mail: johannes.stallhofer@med.uni-jena.de

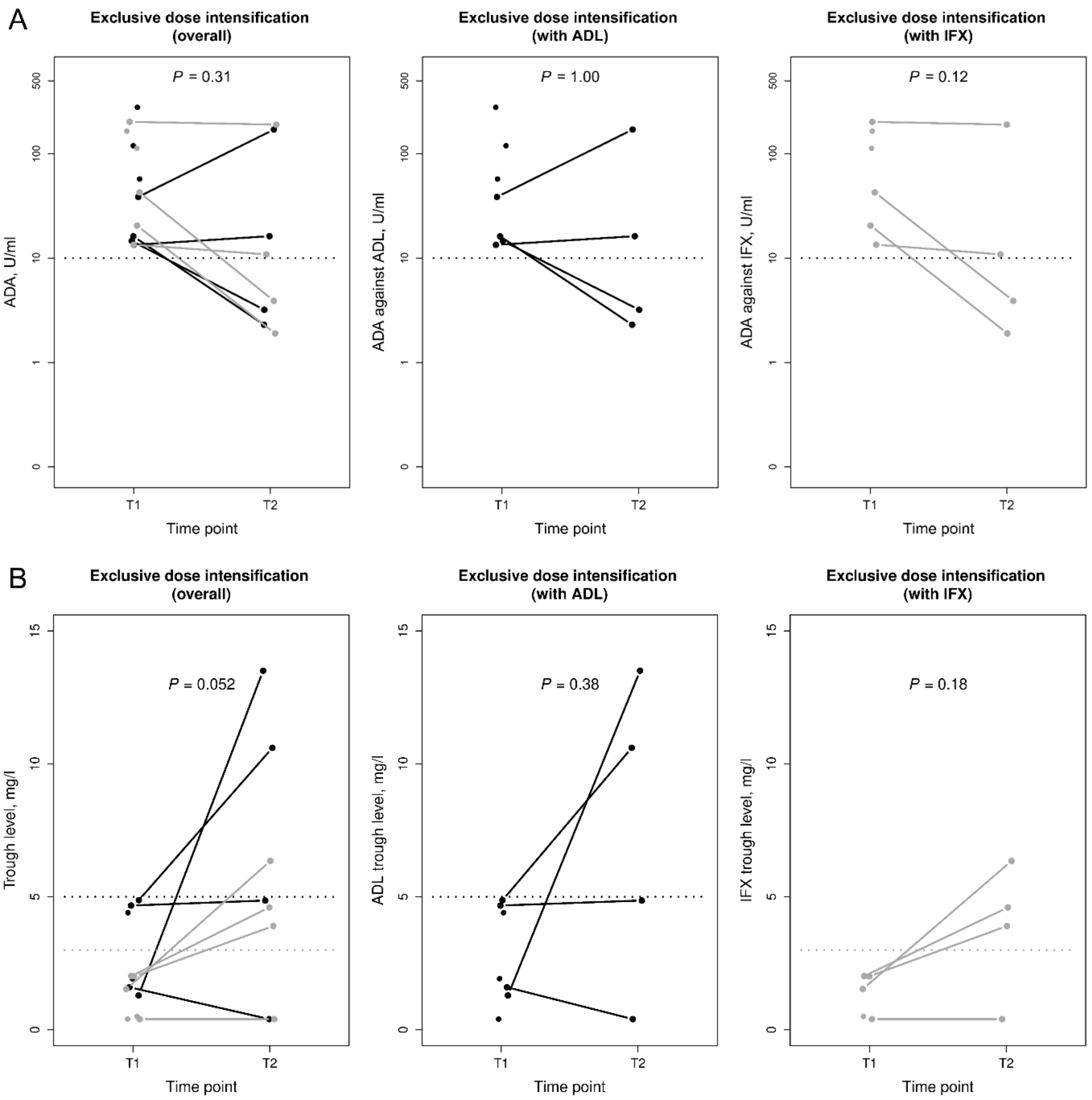

Supplementary Figure 1. Course of anti-drug antibody levels (A) against adalimumab (black) and infliximab (grey) and course of adalimumab (black) and infliximab (grey) trough levels (B) in inflammatory bowel disease patients receiving an exclusive anti-TNF dose intensification following an immunogenic loss of response. *Cutoff values of 5 mg/l for therapeutic adalimumab trough levels and of 3 mg/l for therapeutic infliximab trough levels are marked as horizontal lines. Comparison by Wilcoxon signed rank test. Abbreviations: ADA, anti-drug antibodies; ADL, adalimumab; IFX, infliximab; T1, time point 1 (time of immunogenic loss of therapeutic response); T2, time point 2.*
