# Supplementary material for: Immunomodulator comedication promotes the reversal of anti-drug antibody-mediated loss of response to anti-TNF therapy in inflammatory bowel disease
Source: Int J Colorectal Dis. 2023 Feb 25;38(1):54. doi: 10.1007/s00384-023-04349-1 (PMC9968255; doi:10.1007/s00384-023-04349-1)
Supplement: Supplementary file 3 — Supplementary file3 (PDF 223 KB) [file 384_2023_4349_MOESM3_ESM.pdf]

Online Resource 3

Article: Immunomodulator Comedication Promotes the Reversal of Anti-Drug Antibody-Mediated Loss of Response to Anti-TNF Therapy in Inflammatory Bowel Disease
Journal: International Journal of Colorectal Disease
Authors: Johannes Stallhofer, Jan Guse, Miriam Kesselmeier, Philip Christian Grunert, Kathleen Lange, Robert Stalmann, Verena Eckardt, Andreas Stallmach

Corresponding author: Dr. med. Johannes Stallhofer, Jena University Hospital, Department of Internal Medicine IV, E-mail: johannes.stallhofer@med.uni-jena.de

Supplementary Table 2. Diagnostic capacity of anti-drug antibody levels, TNF inhibitor trough levels and C-reactive protein levels at timepoint T1 (time of immunogenic loss of response) to predict the success of an immunomodulator combination therapy or the failure of an exclusive anti-TNF dose intensification concerning the three endpoints regain of clinical remission, anti-drug antibody clearance and regain of clinical remission along with pharmacokinetic response.

Intervention	Measure	Endpoint	AUC (95 % CI)	Optimal cutoff (Youden)	TP	FP	TN	FN	Sensitivity	Specificity	PPV	NPV
Immunomodulator combination therapy	ADA titer, U/ml	Regain of clinical remission	0.63 (0.26, 1.00)	<433.00	17	2	1	0	1.00	0.33	0.89	1.00
		ADA clearance	0.61 (0.32, 0.89)	<22.50	5	0	6	9	0.36	1.00	1.00	0.40
		Regain of clinical remission along with ADA clearance	0.70 (0.46, 0.94)	<22.50	5	0	9	6	0.45	1.00	1.00	0.60
	Trough level, mg/l	Regain of clinical remission	0.86 (0.68, 1.00)	≥0.98	12	0	3	5	0.71	1.00	1.00	0.38
		ADA clearance	0.87 (0.71, 1.00)	≥1.00	11	0	6	3	0.79	1.00	1.00	0.67
		Regain of clinical remission along with ADA clearance	0.69 (0.46, 0.93)	≥1.40	7	2	7	4	0.64	0.78	0.78	0.64
	CRP, mg/l	Regain of clinical remission	0.76 (0.43, 1.00)	<38.40	16	1	2	1	0.94	0.67	0.94	0.67
		ADA clearance	0.80 (0.56, 1.00)	<38.40	14	3	3	0	1.00	0.50	0.82	1.00
		Regain of clinical remission along with ADA clearance	0.65 (0.40, 0.90)	<38.40	11	6	3	0	1.00	0.33	0.65	1.00
Exclusive anti-TNF dose intensification	ADA titer, U/ml	No regain of clinical remission	0.77 (0.46, 1.00)	≥38.60	8	0	2	3	0.73	1.00	1.00	0.40
		No ADA clearance	0.75 (0.47, 1.00)	≥57.36	6	0	4	3	0.67	1.00	1.00	0.57
		No regain of clinical remission along with ADA clearance	0.77 (0.46, 1.00)	≥38.60	8	0	2	3	0.73	1.00	1.00	0.40
	Trough level, mg/l	No regain of clinical remission	0.50 (0.05, 0.95)	<1.29	4	0	2	7	0.36	1.00	1.00	0.22
		No ADA clearance	0.75 (0.43, 1.00)	<2.01	7	1	3	2	0.78	0.75	0.88	0.60
		No regain of clinical remission along with ADA clearance	0.50 (0.05, 0.95)	<1.29	4	0	2	7	0.36	1.00	1.00	0.22
	CRP, mg/l	No regain of clinical remission	0.89 (0.68, 1.00)	≥6.00	9	0	2	2	0.82	1.00	1.00	0.50
		No ADA clearance	0.81 (0.56, 1.00)	≥6.00	8	1	3	1	0.89	0.75	0.89	0.75
		No regain of clinical remission along with ADA clearance	0.89 (0.68, 1.00)	≥6.00	9	0	2	2	0.82	1.00	1.00	0.50

Abbreviations: ADA, anti-drug antibodies; AUC, area under the curve; CI, confidence interval; CRP, C-reactive protein; FN, false negative; FP, false positive; NPV, negative predictive value; PPV, positive predictive value; TN, true negative; TP, true positive.

Supplementary Table 3. Diagnostic capacity of different combinations of anti-drug antibody levels, TNF inhibitor trough levels and C-reactive protein levels at timepoint T1 (immunogenic loss of response) to predict the success of an immunomodulator combination therapy or a failure of an exclusive anti-TNF dose intensification concerning the three endpoints regain of clinical remission, anti-drug antibody clearance and regain of clinical remission along with pharmacokinetic response. *Cutoff values were deduced from the results presented in Supplementary Table 1.*

Intervention	Combination	Condition 1	Condition 2	Endpoint	TP	FP	TN	FN	Sensitivity	Specificity	PPV	NPV
Immunomodulator combination therapy	ADA level or trough level	ADA level, U/ml <433.00	Trough level, mg/l ≥0.98	Regain of clinical remission	17	2	1	0	1.00	0.33	0.89	1.00
		ADA level, U/ml <22.50	Trough level, mg/l ≥1.00	ADA clearance	11	0	6	3	0.79	1.00	1.00	0.67
		ADA level, U/ml <22.50	Trough level, mg/l ≥1.40	Regain of clinical remission along with ADA clearance	8	2	7	3	0.73	0.78	0.80	0.70
	ADA level or CRP	ADA level, U/ml <433.00	CRP, mg/l <38.40	Regain of clinical remission	17	3	0	0	1.00	0.00	0.85	-
		ADA level, U/ml <22.50	CRP, mg/l <38.40	ADA clearance	14	3	3	0	1.00	0.50	0.82	1.00
		ADA level, U/ml <22.50	CRP, mg/l <38.40	Regain of clinical remission along with ADA clearance	11	6	3	0	1.00	0.33	0.65	1.00
	Trough level or CRP	Trough level, mg/l ≥0.98	CRP, mg/l <38.40	Regain of clinical remission	16	1	2	1	0.94	0.67	0.94	0.67
		Trough level, mg/l ≥1.00	CRP, mg/l <38.40	ADA clearance	14	3	3	0	1.00	0.50	0.82	1.00
		Trough level, mg/l ≥1.40	CRP, mg/l <38.40	Regain of clinical remission along with ADA clearance	11	6	3	0	1.00	0.33	0.65	1.00
Exclusive anti-TNF dose intensification	ADA level or trough level	ADA level, U/ml ≥38.60	Trough level, mg/l <1.29	No regain of clinical remission	8	0	2	3	0.73	1.00	1.00	0.40
		ADA level, U/ml ≥57.36	Trough level, mg/l <2.01	No ADA clearance	8	1	3	1	0.89	0.75	0.89	0.75
		ADA level, U/ml ≥38.60	Trough level, mg/l <1.29	No regain of clinical remission along with ADA clearance	8	0	2	3	0.73	1.00	1.00	0.40
	ADA level or CRP	ADA level, U/ml ≥38.60	CRP, mg/l ≥6.00	No regain of clinical remission	11	0	2	0	1.00	1.00	1.00	1.00
		ADA level, U/ml ≥57.36	CRP, mg/l ≥6.00	No ADA clearance	9	1	3	0	1.00	0.75	0.90	1.00
		ADA level, U/ml ≥38.60	CRP, mg/l ≥6.00	No regain of clinical remission along with ADA clearance	11	0	2	0	1.00	1.00	1.00	1.00
	Trough level or CRP	Trough level, mg/l <1.29	CRP, mg/l ≥6.00	No regain of clinical remission	9	0	2	2	0.82	1.00	1.00	0.50
		Trough level, mg/l <2.01	CRP, mg/l ≥6.00	No ADA clearance	8	2	2	1	0.89	0.50	0.80	0.67
		Trough level, mg/l <1.29	CRP, mg/l ≥6.00	No regain of clinical remission along with ADA clearance	9	0	2	2	0.82	1.00	1.00	0.50

Abbreviations: ADA, anti-drug antibodies; CRP, C-reactive protein; FN, false negative; FP, false positive; NPV, negative predictive value; PPV, positive predictive value; TN, true negative; TP, true positive.
