# Supplementary material for: Immunomodulator comedication promotes the reversal of anti-drug antibody-mediated loss of response to anti-TNF therapy in inflammatory bowel disease
Source: Int J Colorectal Dis. 2023 Feb 25;38(1):54. doi: 10.1007/s00384-023-04349-1 (PMC9968255; doi:10.1007/s00384-023-04349-1)
Supplement: Supplementary file 4 — Supplementary file4 (PDF 73 KB) [file 384_2023_4349_MOESM4_ESM.pdf]

Online Resource 4

Article: Immunomodulator Comedication Promotes the Reversal of Anti-Drug Antibody-Mediated Loss of Response to Anti-TNF Therapy in Inflammatory Bowel Disease
Journal: International Journal of Colorectal Disease
Authors: Johannes Stallhofer, Jan Guse, Miriam Kesselmeier, Philip Christian Grunert, Kathleen Lange, Robert Stalmann, Verena Eckardt, Andreas Stallmach

Corresponding author: Dr. med. Johannes Stallhofer, Jena University Hospital, Department of Internal Medicine IV, E-mail: johannes.stallhofer@med.uni-jena.de

Supplementary Text

High ADA titers in unsuccessful dose intensification attempts

1. Descriptive summary for IBD patients on adalimumab and infliximab

Of seven patients, who underwent a dose intensification of ADL alone, three (43 %) had to stop this attempt due to a significant IBD flare already after a median of two (one, four) weeks. These three patients had a high median ADA level against ADL of 119.50 (57.36, 279.10) U/ml. Furthermore, in two out of seven patients (29 %) with ADA against ADL of 38.60 U/ml and 13.40 U/ml, dose intensification did not result in a restoration of therapeutic trough levels ≥ 5 mg/l. In both cases, dose intensification even led to an increase in the ADA levels to 171.00 U/ml and 16.24 U/ml after seven and three months, respectively. Only two of the seven patients (29 %) with the lowest ADA titers against ADL of 14.62 U/ml and 16.18 U/ml were able to regain a therapeutic trough level ≥ 5 mg/l together with negative ADA < 10 U/ml after eight and 23 months, respectively; one of them responded clinically. The remaining five out of seven IBD patients (71 %), in whom dose intensification of ADL failed pharmacokinetically, had nominally higher ADA titers (median 57.36 [26.00, 199.30] U/ml) than the two of seven (29 %), in whom this attempt was pharmacokinetically successful (P = 0.38).

Similarly, of six patients, in whom a dose intensification of the IFX regimen was tried to overcome LOR, two (33 %) experienced a disease flare, which led the treating physician to switch therapy after four and seven weeks. These two patients yet again had high ADA levels against IFX of 113.00 U/ml and 164.90 U/ml, and only received one additional infusion of IFX after the documented pharmacokinetic and clinical LOR. Of the six patients, in an additional two of six patients (33 %) with ADA levels against IFX of 13.47 U/ml and 202.60 U/ml, ADA could not be suppressed to < 10 U/ml after two and six months; these two ADA levels both remained on about the same level with 10.86 U/ml and 190.50 U/ml, respectively. Again, only the remaining two out of six IFX-treated patients (33 %) with low ADA levels against IFX of 42.70 U/ml and 20.50 U/ml were able to restore therapeutic trough levels ≥ 3 mg/l and to completely resolve ADA after two and 21 months, respectively; one of them regained clinical remission. Just like in the ADL-treated group, the four of the six IFX-treated patients (66 %) with a failure of the dose intensification attempt concerning pharmacokinetic response had nominally higher ADA levels of median 138.95 (38.35, 193.18) U/ml than the two of the six (33 %) pharmacokinetic responders (P = 0.53).

2. Descriptive summary for patients with ulcerative colitis and Crohn's disease

Of the three out of nine UC patients (two on ADL, one on IFX), who were administered a dose intensification, two (1 on ADL, 1 on IFX; 66 %) showed a complete pharmacokinetic response and regain of clinical remission after eight and 21 months. These two patients were the only combined pharmacokinetic and clinical responders to dose intensification alone in our entire cohort. However, these two patients had particularly low ADA titers of 14.62 U/ml against ADL and 20.50 U/ml against IFX. In the one remaining patient (33 %), ADL had to be stopped due to a clinically significant disease flare after two weeks and only two additional doses of ADL.

Of the ten out of 24 CD patients (five on ADL, five on IFX), who got a dose intensification as the only therapeutic strategy, none regained complete pharmacokinetic response and clinical remission. A complete clearance of ADA and therapeutic trough levels could only be detected for two out of 10 patients (20 %; one on ADL, one on IFX) after 23 and two months, but without clinical remission. A clinical remission could not be observed for any of the ten CD patients (0/10 patients, 0 %) with dose intensification only.

3. Combined data set

When combining the dataset of ADL-treated and IFX-treated IBD patients, an exclusive anti-TNF dose intensification attempt in order to overcome an immunogenic LOR showed at least a pharmacokinetic response in four out of 13 patients (31 %). However, only two of these four patients with a reconstitution of therapeutic drug levels and clearance of ADA responded with clinical remission to this strategy. Nine out of

13 patients (69 %) did not regain clinical remission and only four of these nine patients had a second ADA and trough level measurement. These nine patients displayed a nominally higher initial median ADA level of 113.00 (26.04, 183.75) U/ml compared to the four pharmacokinetic responders with a median ADA level against ADL or IFX of 18.34 (15.01, 37.15) U/ml at the time of LOR (P = 0.20). The two out of 13 clinical responders (15 %) to the dose intensification attempt, who regained clinical remission along with pharmacokinetic response, had particularly low ADA levels of 14.62 U/ml and 20.50 U/ml compared to a median ADA level of 57.36 (16.18, 164.90) U/ml in the eleven out of 13 non-responders (85 %; P = 0.31).
